# Impact of the COVID-19 Pandemic on the Everyday Life and Healthcare of Patients with Congenital Heart Defects: Insights from Pandemic Onset to One Year Later

**DOI:** 10.3390/jcm14103462

**Published:** 2025-05-15

**Authors:** Emily Schütte, Saskia Olivia Nasri, Anna-Lena Ehmann, Janina Semmler, Felix Berger, Ulrike M. M. Bauer, Katharina Schmitt, Cornelia Tremblay, Julia Remmele, Stefan Orwat, Gerhard-Paul Diller, Constanze Pfitzer, Paul C. Helm

**Affiliations:** 1 Deutsches Herzzentrum der Charité, Department of Congenital Heart Disease—Pediatric Cardiology, Augustenburger Platz 1, 13353 Berlin, Germany; emily.schuette@charite.de (E.S.); olivia.nasri@gmx.de (S.O.N.); anna-lena.ehmann@charite.de (A.-L.E.); felix.berger@dhzc-charite.de (F.B.); katharina.schmitt@dhzc-charite.de (K.S.); 2 Charité—Universitätsmedizin Berlin, Corporate Member of Freie Universität Berlin and Humboldt-Universität zu Berlin, Charitéplatz 1, 10117 Berlin, Germany; 3 National Register for Congenital Heart Defects, Augustenburger Platz 1, 13353 Berlin, Germany; ulrike.bauer@dhzc-charite.de (U.M.M.B.); cornelia.tremblay@charite.de (C.T.); paul.helm@dhzc-charite.de (P.C.H.); 4 Department of Obstetrics, Charité-Universitätsmedizin Berlin, Corporate Member of Freie Universität Berlin and Humboldt-Universität zu Berlin, Augustenburger Platz 1, 13353 Berlin, Germany; janina.semmler@charite.de; 5 Competence Network for Congenital Heart Defects, Augustenburger Platz 1, 13353 Berlin, Germany; julia.remmele@tum.de (J.R.); gerhard.diller@ukmuenster.de (G.-P.D.); 6 Deutsches Herzzentrum der Charité, Department of Psycho-Cardiology, Augustenburger Platz 1, 13353 Berlin, Germany; 7 Department of Congenital Heart Disease and Pediatric Cardiology, German Heart Center of Munich, Georg Brauchle Ring 62, 80992 Munich, Germany; 8 Institute of Preventive Pediatrics Technical University Munich, Georg Brauchle Ring 62, 80992 Munich, Germany; 9 Adult Congenital and Valvular Heart Disease Center, Department of Cardiology and Angiology, University Hospital Muenster, Albert-Schweitzer-Campus 1, 48149 Muenster, Germany; orwat@ukmuenster.de

**Keywords:** congenital heart defects, COVID-19, health care utilization, everyday life, risk perception

## Abstract

**Background/Objectives:** The COVID-19 pandemic impacted healthcare access globally, with chronic conditions like congenital heart defects (CHD) posing unique challenges. While general trends have been studied, little is known about the impact on CHD patients in Europe. This study assessed the living conditions, healthcare utilization, and psychosocial well-being of CHD patients and their families in Germany, considering CHD severity, gender differences, and changes over time. **Methods:** Data were derived from two cross-sectional online surveys conducted by the National Register for Congenital Heart Defects (NRCHD) in April 2020 and April 2021. Surveys targeted CHD patients and relatives, assessing healthcare access, risk perception, COVID-19-related knowledge, and psychosocial effects. Statistical analyses compared responses by gender, CHD severity, and survey period. **Results:** A total of 6737 responses were analyzed. Healthcare utilization declined early in the pandemic due to infection fears but partially recovered in 2021. Perceived health risks increased from 27.9% in 2020 to 38.7% in 2021 (*p* < 0.001), along with higher psychosocial distress. COVID-19-related knowledge was greater in 2020, but trust in government information declined, while reliance on healthcare providers increased. Gender and CHD severity influenced healthcare engagement and perceptions. **Conclusions:** This study highlights the challenges CHD patients faced, underscoring the need for holistic, patient-centered care. Future interventions should focus on tailored communication and support strategies, particularly for vulnerable populations, to mitigate the impact of future health crises.

## 1. Introduction

In 2020, the global COVID-19 pandemic introduced new fears and risks to the general population and posed unprecedented challenges to healthcare systems worldwide [1,2]. In Germany, as in many other countries, the implementation of nationwide measures and laws, aimed at curbing the spread of the Severe Acute Respiratory Syndrome Coronavirus 2 (SARS-CoV-2), had profound impacts on social, societal, and personal life [3,4,5]. Beyond these broad-reaching effects, the pandemic also had a substantial influence on healthcare provision, leading to a decline in the utilization of both outpatient and clinical care [6,7,8].

While the societal and healthcare impacts of the pandemic affected the population as a whole, individuals with chronic diseases were particularly vulnerable to these changes [9,10]. In a cohort study, over 40% of patients with chronic conditions canceled at least one preventive examination and cited fear of contracting COVID-19 as the primary reason [9]. Furthermore, a German population-based study with over 9000 participants revealed that approximately 12% of respondents missed specialist follow-up appointments, while 10% refrained from visiting a doctor despite experiencing symptoms [11]. Additionally, inpatient care changes were observed during the pandemic [12]. For instance, hospitalization rates for patients with cardiovascular diseases in Germany declined significantly between March and September 2020, along with a reduction in heart surgeries [12]. In addition to COVID-19-related postponements by hospitals, fear of infection was cited as a contributing factor [12].

The hesitancy to pursue medical care during the pandemic was also observed among patients with congenital heart defects (CHD), both pediatric and adult [13]. The underlying reasons for this avoidance behavior are complex and have been little studied to date. One study in the United States among CHD patients and their families revealed that 75% of these patients expressed moderate-to-high levels of fear regarding potential COVID-19 infection. Additionally, roughly half of the respondents (50% pediatric, 42% adult) reported experiencing psychological distress during the pandemic. The survey further highlighted an intense desire among CHD patients for more information related to COVID-19, particularly in the context of their condition [13]. However, there is a lack of further studies that have investigated this context, especially in patients with CHD in Europe.

To close this research gap, this study aims to evaluate the living conditions and healthcare situation of CHD patients and their relatives during the COVID-19 pandemic, considering the impact of CHD severity and gender. Additionally, this study seeks to analyze how perceived knowledge about COVID-19 and risk assessment evolved after one year into the pandemic as well as to examine the broader implications of these experiences on their medical care and overall well-being. To the best of our knowledge, this study is the first analyzing this important and complex context.

## 2. Materials and Methods

### 2.1. Study Design and Patient Cohort

The data used for the following statistical analyses were derived from two cross-sectional surveys conducted by the National Register for Congenital Heart Defects (NRCHD) regarding the COVID-19 pandemic. The participants included patients registered with the NRCHD, or their relatives/legal guardians for children, adolescents, or individuals unable to participate independently. Inclusion criteria were NRCHD registration, a diagnosis of CHD regardless of severity, available contact information, and consent for communication from NRCHD. The CHD severity grading is based on the international classification by Warnes et al., following the American College of Cardiology (ACC) recommendations, and is categorized into simple, moderate, and complex [14]. Both surveys were conducted exclusively online, and each survey contained closed questions and a free-text field for individual opinions, allowing for quantitative and qualitative data analysis. In the development of the survey tools and research questions, caregivers and research experts were involved to ensure relevance to patient experiences. Direct patient involvement in the design phase was not feasible due to pandemic restrictions, disproportionately high economic costs and the time pressure caused by the rapid changes in the pandemic situation itself. The questions were informed by patient feedback from clinical practice, research findings, and the German nationwide COSMO longitudinal study [15]. This collaborative approach helped align the study design with best practices in participatory health research.

Survey 1 was conducted anonymously shortly after the pandemic outbreak in April 2020. Participants were contacted via email and invited to complete a questionnaire consisting of 49 questions. In addition to demographic data, three different main areas were surveyed. First, information was collected on underlying health conditions, general health status, and access to healthcare since the pandemic began. Furthermore, participants were asked to evaluate 13 statements regarding their subjective risk perception of COVID-19 on a six-point scale (ranging from “none” to “very high”). Additionally, COVID-19-related knowledge was assessed using 25 statements rated on a seven-point scale (1 = not at all, 7 = fully). The survey took approximately 20 min to complete, with answers provided in binary (yes/no), Likert scales, or multiple-choice formats, and some questions allowed multiple answers. Questions specific to the patient’s perspective were marked as “patient questions”.

Survey 2, conducted in April 2021, included 78 closed-ended questions with formats consistent with Survey 1. Participants were invited via mail or email. In addition to topics from the first survey, new questions related to COVID-19 testing, vaccination, knowledge of protective measures, and experiences with COVID-19 infection were included. This survey took approximately 30 min to complete. As in the first survey, questions intended to be answered from the patient’s perspective were labeled accordingly.

### 2.2. National Register for Congenital Heart Defects

The NRCHD is a scientific organization founded in 2003 by German cardiology societies (DGK, DGPK, and DGTHG). It focuses on research in the field of CHD and maintains a nationwide medical database of CHD patients [16]. The NRCHD provides comprehensive data on the prevalence, disease progression, and life expectancy of CHD patients, offering valuable insights into causes, medical care, and quality of life. As of May 2024, around 60,000 patients and their relatives are registered [17]. Participation is voluntary, typically following diagnosis, and registered individuals are invited to take part in studies and stay informed on the latest research [16,18]. Data collected during research projects are stored according to a data protection framework approved by the Berlin Office for Data Protection. The first survey (April 2020) was conducted anonymously, requiring only general ethical approval from the Charité–Universitätsmedizin Berlin. The second survey (April 2021), conducted with pseudonymized data, received an additional ethics approval (EA2/109/21) from the same institution.

### 2.3. Statistical Analyses

Statistical analyses were performed using IBM SPSS (Version 29). Group comparisons were made based on the respondent (patient or relative), gender, and severity of the CHD, as well as between the two survey time points. Differences were tested for significance using appropriate tests: Chi-square test for nominal data, Mann–Whitney U test for ordinal data, and *t*-test for interval-scaled data, depending on the scale level of the items. A significance level of *p* ≤ 0.05 was set. Given that both surveys are primarily exploratory cross-sectional studies, *p*-values are interpreted as exploratory. No adjustment for multiple testing was made, as the focus of the exploratory analysis was to avoid overlooking relevant group differences, which is crucial in exploratory research. While we acknowledge the risk of Type I errors in exploratory analyses, corrections for multiple comparisons were intentionally not applied in order to preserve the ability to detect potentially meaningful patterns worthy of further investigation [19,20,21,22].

## 3. Results

### 3.1. Study Cohort/Patient Characteristics

A total of 6737 CHD patients/relatives were included in the statistical analyses. Survey 1 included 3558 patients or relatives (53% female), while Survey 2 had 3179 participants (52.6% female). In both surveys, more patients than relatives (usually parents) completed the online questionnaires. Significantly more patients participated in Survey 2 compared to Survey 1 (Survey 1: 57% patients; Survey 2: 66.2% patients; *p* < 0.001). Patients in Survey 2 were significantly older, with an average age of 26 years compared to 23.7 years in Survey 1 (*p* < 0.001). Most patients lived in the former West German states, with no significant differences in residential location across the two surveys. However, respondents in Survey 1 were more likely to live in smaller towns or cities with populations of 5000 or less (Survey 1: 31.1%; Survey 2: 22.1%; *p* < 0.001). There were also significant differences in educational attainment and employment status between the two surveys (*p* < 0.001), particularly among those who were not yet in school. Gender differences were also observed, with female patients more likely to complete the questionnaire themselves (Survey 1: 64.4%; Survey 2: 71.3%), while male patients were younger and more likely to work full-time (male: 28.3%; female: 22%). These trends were consistent across both surveys. Further details can be found in Table 1.

### 3.2. Medical and Disease-Related Patient Data

There were significant differences in CHD severity between Survey 1 and Survey 2 (*p* < 0.001). Simple CHD were the least represented, with 25.8% at the beginning of the pandemic and 14.2% in the following year. The proportion of simple CHD significantly decreased from Survey 1 to Survey 2 (*p* < 0.001), while moderate and complex CHD increased (moderate CHD: 36.3% in Survey 1 vs. 39.1% in Survey 2; complex CHD: 30.5% in Survey 1 vs. 37.7% in Survey 2; *p* < 0.001).

Compared to 2020, significantly more patients reported visiting a cardiology practice in Survey 2 (24.4% vs. 39.8%; *p* < 0.001), and twice as many had visited a cardiologist in a hospital or heart center (23.1% vs. 46.6%; *p* < 0.001). Additionally, in 2021, a greater number of patients indicated that they had received hospital treatment since the start of the pandemic (14.3% vs. 5%; *p* < 0.001). In both surveys, only a small proportion of the cohort stated that they themselves (9% in Survey 1 vs. 10.7% in Survey 2) or the doctor or hospital (11.6% in Survey 1 vs. 12.5% in Survey 2) had canceled a check-up/cardiac catheter examination/operation due to COVID-19.

There were also significant increases in the number of patients who had discussions about COVID-19 with their doctors between the two surveys. Significantly more respondents in Survey 2 reported contacting their doctor by phone regarding COVID-19 and their CHD (30% vs. 21.6%; *p* < 0.001), and more received answers from their doctor on these concerns in Survey 2 (46.3% vs. 25.2%; *p* < 0.001).

Furthermore, the proportions of patients vaccinated against influenza differed significantly between the two time points (50.9% in Survey 1 vs. 58.5% in Survey 2; *p* < 0.001), with a larger proportion of respondents in Survey 2 citing COVID-19 as the reason for their influenza or pneumococcal vaccination (9.7% in Survey 2 vs. 4.9% in Survey 1; *p* < 0.001). Further details on medical and disease-related data can be found in Table 2.

### 3.3. Risk Perception

The evaluation of risk perception regarding health, social life, and profession/education revealed significant differences between the first and second survey. At the start of the pandemic, 27.9% of participants in Survey 1 perceived their personal health risk from COVID-19 as high or very high, significantly increasing to 38.7% in Survey 2 (*p* < 0.001). In Survey 2, significantly more patients (42.9%) than relatives (30.4%) rated their personal health risk as high or very high (*p* < 0.001). A significant increase in the perception of risk to family members’ health (40.8% in Survey 2 vs. 20.7% in Survey 1; *p* < 0.001) as well as to the health of close contacts (26.9% in Survey 2 vs. 13.3% in Survey 1; *p* < 0.001) was also observed.

Regarding the perceived risk to their social life, 36.5% of respondents in Survey 2 rated the risk to their social life as high or very high, significantly higher than the 27.1% in Survey 1 (*p* < 0.001). In Survey 1, patients perceived the risk to their social life as lower compared to relatives (25.3% vs. 29.6%; *p* < 0.001), but no significant differences were found in Survey 2. The risk to the social life of respondents’ families and close contacts was also rated significantly higher in the second survey compared to Survey 1 (*p* < 0.001).

Concerning the risk to their own profession or education, 25.8% of participants in Survey 1 assessed it as high or very high, which decreased to 23.8% in Survey 2 (*p* < 0.001). The perception of risk for family members’ education or profession showed an opposite trend, with a higher rating in Survey 2 (24.8%) compared to Survey 1 (19.1%; *p* < 0.001), as did the perceived risk for close contacts (23.4% vs. 14.9%; *p* < 0.001).

**Figure 1:**
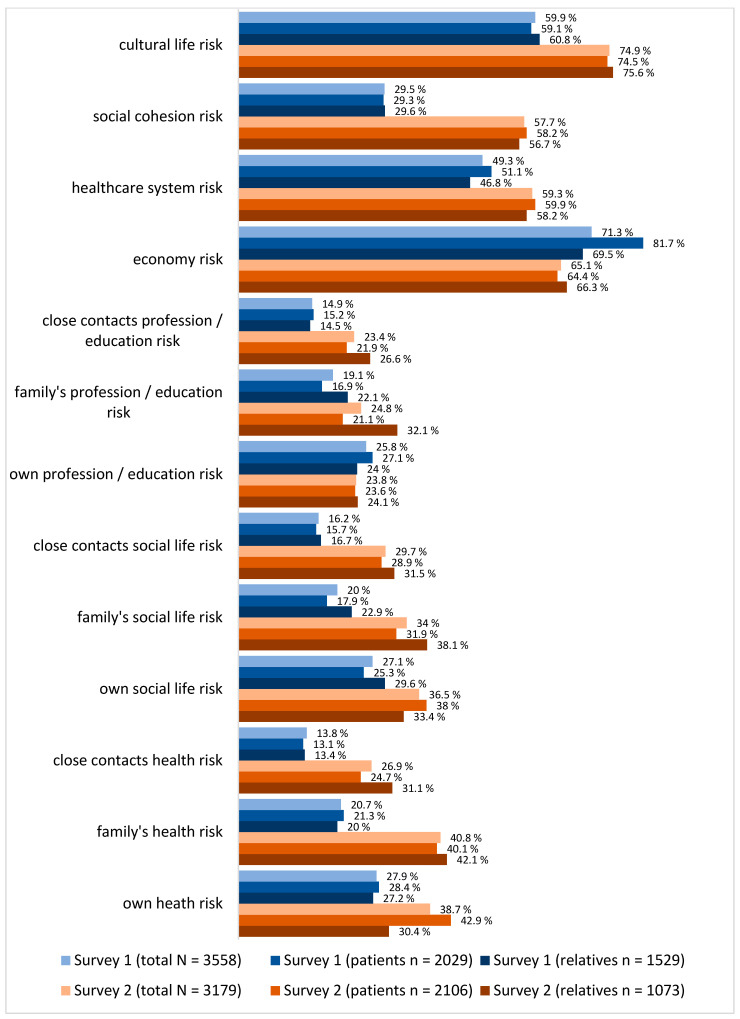
Risk perception.

Furthermore, the majority of respondents in both surveys perceived the risk to the economy and employment as high or very high (71.3% in Survey 1 vs. 65.1% in Survey 2; *p* < 0.001). The risk to the healthcare system was rated as high or very high by 49.3% of respondents in Survey 1, increasing to 59.3% in Survey 2 (*p* < 0.001). More participants in Survey 2 (57.7%) perceived a high or very high risk to social cohesion compared to 29.5% in Survey 1 (*p* < 0.001). Similarly, concerns about the impact on cultural life were significantly higher in Survey 2 (74.9%) than in Survey 1 (59.9%; *p* < 0.001). The visualization of the results is shown in Figure 1. Further details are presented in Table S1 in the Supplementary Material.

### 3.4. COVID-19-Related Knowledge

In the assessment of COVID-19-related knowledge, 19 out of 25 items displayed significant differences between the two survey periods (*p* < 0.001). Participants rated their COVID-19-related knowledge significantly higher at the beginning of the pandemic than in the following year (6.4 ± 1.0 vs. 6.3 ± 0.9; *p* < 0.001), with patients showing higher ratings than relatives in Survey 1 (6.5 ± 0.8 vs. 6.3 ± 1.2; *p* < 0.001). Additionally, participants felt better informed about COVID-19 in the initial survey compared to the subsequent survey (6.2 ± 1.0 vs. 5.9 ± 1.0; *p* < 0.001). In Survey 2, relatives rated their subjective information level higher than patients (6 ± 1.0 vs. 5.9 ± 1.1; *p* < 0.001).

In Survey 1, participants were significantly more likely to report following daily media coverage (Internet/TV/Radio/Newspaper) about COVID-19 than those in Survey 2 (5.6 ± 1.7 vs. 5 ± 1.9; *p* < 0.001). They also rated their use of social media for COVID-19 information (3.9 ± 2.1 vs. 3.3 ± 2.1; *p* < 0.001) as well as their trust in official government information (5.5 ± 1.4 vs. 4.9 ± 1.7; *p* < 0.001) significantly higher at the beginning of the pandemic.

However, in Survey 2, participants felt significantly more informed by their doctor about COVID-19 (4.94 ± 1.8 vs. 4.3 ± 2.1; *p* < 0.001). The largest difference was seen in the likelihood of avoiding doctor visits due to COVID-19, with Survey 1 participants significantly more inclined to avoid visits compared to those in Survey 2 (4.5 ± 2.1 vs. 3.7 ± 2.2; *p* < 0.001).

Both surveys also revealed significant differences between patients and relatives. The most notable difference emerged in the question of whether participants would consult their doctor for questions regarding their CHD or COVID-19, with relatives showing significantly higher agreement than patients in both surveys. More detailed information is provided in Table 3.

## 4. Discussion

This study provides significant insights into the impact of the COVID-19 pandemic on patients with CHD and their relatives in Germany, highlighting both the direct and indirect effects on healthcare utilization, risk perception, and knowledge surrounding COVID-19. Data from a total of 6737 responses were gathered from patients with CHD and their relatives through two nationwide exploratory online surveys conducted in April 2020 and April 2021. The results reveal considerable shifts in the health behavior and attitudes of CHD patients and their relatives, as well as notable differences between the initial stages of the pandemic and one year later. To interpret the findings appropriately, it is important to contextualize this study within the broader public health measures in place at the time. The first survey (April 2020) coincided with Germany’s initial lockdown phase, including school and business closures, contact restrictions, and widespread uncertainty regarding virus transmission and protection. Public communication was evolving, with daily updates from the Robert Koch Institute (RKI) and increasing emphasis on hygiene and distancing, though mask mandates had not yet been universally implemented. The second survey (April 2021) occurred in a different phase, characterized by extended restrictions, partial reopening strategies, the introduction of vaccines, and growing pandemic fatigue. These contextual differences likely shaped changes in risk perception, healthcare behavior, and emotional responses observed in the present study.

Participants were broadly representative of Germany, with responses from all regions, although slightly more participants were from the former West German states. Additionally, significantly more patients completed the survey themselves in 2021 compared to 2020. This increase in patient participation could be linked to the predominance of adult CHD patients (ACHD) in Germany, as well as to a heightened need for these patients to express their healthcare concerns amidst pandemic-induced pressures on the healthcare system [23].

One of the most prominent findings is the significant increase in perceived health risks associated with COVID-19 between the two surveys. At the start of the pandemic, participants generally assessed their personal and familial health risks as lower, but these perceptions intensified in Survey 2. This shift may reflect the evolving pandemic context in Germany, where escalating case numbers and ongoing media coverage could have amplified public awareness and concern [24]. Notably, in Survey 2, patients themselves were more likely than relatives to perceive a high risk to their own health, a trend consistent with findings from other chronic disease populations, where increased self-perceived vulnerability to COVID-19 has been documented [13,25]. Our findings are partially consistent with national and European surveys of the general population’s response to COVID-19. A comparison with studies on the general population in Germany reveals both overlaps and distinctions in risk perception during the first pandemic year. Similar to CHD patients, the broader public reported significantly higher perceived health risks by April 2021 than at the pandemic’s onset. Early confidence in isolation measures decreased over time, likely influenced by intensified media coverage and rising infection numbers—confirmed by the RKI [26,27].

Another important observation is the increased perception of risk to social life and economic stability over time. In Survey 1, participants primarily focused on health risks; by Survey 2, however, emphasis had shifted toward the societal and economic ramifications of the pandemic. This progression in risk perception mirrors findings from other studies, which indicated that social restrictions and isolation were primary concerns for many people as the pandemic persisted [28,29]. The Mannheim Corona Study showed that most participants resumed near-normal contact behavior by May 2020, while individuals with a stronger perceived threat—mirroring patterns in the CHD group—avoided private gatherings [30]. Therefore, for patients with CHD, who may already experience social limitations due to their condition [31], the prolonged nature of social restrictions imposed additional burdens on their mental and emotional well-being [25]. Interestingly, while general economic concerns decreased slightly in our study (from over 70% to 65.1%), fear regarding the healthcare system increased (from 49.3% to 59.3%), likely reflecting broader anxieties about system capacity despite regional restrictions and is in line with results presented by the German Federal Statistical Office [32]. These findings support the observed trend in CHD patients and underscore the need for differentiated communication and support strategies for high-risk groups. These comparisons reveal that, while some concerns mirrored those of the general population, CHD patients exhibited consistently elevated healthcare-related anxieties and social isolation, underscoring the need for tailored responses.

Regarding the healthcare utilization, the data also indicated a decrease in outpatient and hospital visits among CHD patients during the initial months of the pandemic. Fear of COVID-19 infection likely contributed to this decline, as evidenced by the high proportion of participants in Survey 1 who reported avoiding medical appointments due to concerns about contracting the virus. This hesitancy aligns with broader population trends seen in Germany and globally, where chronic disease patients have been shown to defer routine healthcare visits due to pandemic-related anxieties [9,10]. However, in Survey 2, an increase in cardiology and hospital visits suggests that initial fears may have lessened as understanding and adaptation to COVID-19 evolved, though it is unclear to what extent delayed care may have impacted health outcomes in this cohort [33].

This study also highlights significant differences in COVID-19-related knowledge between the two survey periods. Initial assessments in Survey 1 showed a higher self-rated knowledge level, likely due to the intense media focus on the pandemic’s onset. Interestingly, trust in governmental information was higher at the pandemic’s outset than a year later, suggesting a potential erosion of confidence in official sources as the pandemic progressed. This shift in trust might reflect a growing reliance on personal healthcare providers for information, as indicated by the increased engagement with healthcare professionals observed in Survey 2. The finding aligns with studies that emphasize the critical role of healthcare providers in patient education during health crises [13,34,35].

The gender differences observed in healthcare engagement and risk perception provide additional insight. Female CHD patients were more likely than their male counterparts to complete the surveys independently, reflecting a potential gender-based discrepancy in health-seeking behavior or perceived need for information. Such disparities underscore the importance of targeted communication and support strategies considering gender differences in health behavior and information needs [36].

Lastly, this study’s findings reveal substantial gaps in psychosocial support for CHD patients, who reported high levels of psychological distress during the pandemic. While medical care remained a priority, there was an expressed need for more precise guidance on managing pandemic-related fears, navigating isolation, and understanding the specific risks posed by COVID-19 to individuals with CHD. These needs emphasize the value of a holistic approach to healthcare, especially in times of crisis, where psychosocial factors can heavily influence health outcomes and quality of life.

### Limitations

The limitations of this study must be considered when interpreting these results. One of the main methodological constraints is the use of two independent cross-sectional surveys rather than a longitudinal design. This approach restricts our ability to draw conclusions about intra-individual changes in healthcare experiences and psychosocial impacts over time. However, a longitudinal design was not feasible within the scope and logistics of our data collection phases, particularly during the rapidly evolving pandemic context. Nevertheless, our two time points for data collection were selected to reflect key phases of the pandemic, offering a population-level perspective on changing perceptions. These cross-sectional snapshots offer valuable insights into how perceptions, behaviors, and needs evolved over the course of the pandemic among patients with CHD and their families.

Secondly, we acknowledge that reliance on self-reported data introduces potential biases, including recall inaccuracies and socially desirable responses. To minimize these biases, the surveys were carefully designed with structured, non-leading questions, and participants were assured of the anonymity of their responses.

The urgency of data collection during the early pandemic phases prioritized rapid deployment over formal psychometric validation. Therefore, no standardized instruments for measuring mental stress, such as the PHQ-9 or GAD-7, were used, a fact that should be considered. Instead, psychosocial concerns were assessed through questions based on themes reported during the early stages of the pandemic in the media, research, and clinical practice.

Furthermore, the findings of this study are influenced by the specific structure and characteristics of Germany’s healthcare system, particularly in the context of access to care during the pandemic. While this may limit the generalizability of our results to other healthcare systems, it can be assumed that our findings remain relevant for countries with comparable healthcare models or those facing similar chronic care challenges.

We also recognize the limitation of using an exclusively online survey format, which may have excluded certain subgroups, such as older adults or individuals with limited digital access. The online approach was chosen in response to pandemic-related restrictions and allowed for rapid data collection across a large geographic area. This being said, online surveys are state of the art.

Finally, participation varied significantly across comparison groups, particularly between patients and relatives, which may limit the strength of conclusions based on these comparisons. Furthermore, due to the absence of data on the educational background of invited individuals, it is not possible to assess whether education influenced participation rates. However, the overall sample sizes (3558 participants in Survey 1 and 3179 in Survey 2) are substantial enough to provide a reliable view of the life and treatment situation of CHD patients and their families.

## 5. Conclusions

Overall, this study underscores the multifaceted impact of the COVID-19 pandemic on CHD patients and their relatives, affecting not only healthcare access and utilization but also risk perception, knowledge, and psychosocial well-being. The results highlight a critical need for tailored support and comprehensive healthcare strategies that address both medical and psychosocial dimensions, particularly for vulnerable patient populations. Future research should consider longitudinal designs to better understand the long-term effects of future healthcare crisis on CHD patients. However, these results provide an important first basis and should be considered when developing interventions to mitigate similar challenges in future health crises.

## Figures and Tables

**Table 1:** Sample description.

		Survey 1 (April 2020)			Survey 2 (April 2021)		
		Total N = 3558	Male n = 1670 *	Female n = 1886 *	Total N = 3179	Male n = 1505 **	Female n = 1674 **
**Participants**	Patient	57% ^a^	48.7% ^d^	64.4% ^d^	66.2% ^a^	60.7% ^d^	71.3% ^d^
	Relative	43% ^a^	51.3% ^d^	35.6% ^d^	33.8% ^a^	39.3% ^d^	28.7% ^d^
**Patient age in years (mean ± SD)**		23.7 ± 15.4 ^a^	22.4 ± 16 ^d^	24.7 ± 14.9 ^d^	26 ± 16.1 ^a^	25.6 ± 16.9	26.4 ± 15.3
**Federal state**	Old federal states	69.6%	70.6%	68.7%	68.0%	68.1%	68.0%
	New federal states	28.9%	28.1%	29.6%	30.9%	30.8%	30.9%
	Unknown/Foreign country	1.5%	1.3%	1.7%	1.1%	1.1%	1.1%
**Residence size**	5000 inhabitants or less	31.1% ^a^	31.3%	31.2%	22.1% ^a^	21.5%	22.5%
	5001–20,000 inhabitants	21.4% ^a^	21.3%	21.4%	25.8% ^a^	26.4%	25.3%
	20,001–100,000 inhabitants	18.8% ^a^	18.4%	19.2%	21.2% ^a^	20.8%	21.6%
	More than 100,000 inhabitants	28.4% ^a^	28.8%	28.0%	30.9% ^a^	31.3%	30.6%
	I do not know	0.2% ^a^	0.2%	0.2%	-	-	-
**Highest educational qualification**	I don’t go to school yet	14.8% ^a^	17.2% ^d^	12.7% ^d^	11.0% ^a^	12. 9% ^d^	9.3% ^d^
	Finished school without a degree	2.0% ^a^	2.2% ^d^	1.9% ^d^	1.8% ^a^	2.0% ^d^	1.6% ^d^
	Still go to school	23.9% ^a^	28.4% ^d^	20.0% ^d^	21.5% ^a^	23. 7% ^d^	19.6% ^d^
	Secondary school certificate ^A^	3.0% ^a^	3.1% ^d^	2.9% ^d^	2.7% ^a^	2.9% ^d^	2.6% ^d^
	Secondary school certificate ^B^	7.8% ^a^	6.8% ^d^	8.6% ^d^	8.6% ^a^	6.8% ^d^	10.2% ^d^
	Completed apprenticeship	11.2% ^a^	8.7% ^d^	13.4% ^d^	12.8% ^a^	12.0% ^d^	13.5% ^d^
	Advanced technical college entrance qualification	5.6% ^a^	5.1% ^d^	6.0% ^d^	6.4% ^a^	5.5% ^d^	7.2% ^d^
	High school diploma	11.0% ^a^	9.5% ^d^	12.5% ^d^	11.7% ^a^	11.3% ^d^	12.1% ^d^
	Technical/university degree	17.0% ^a^	16.2% ^d^	17.8% ^d^	18.8% ^a^	18.1% ^d^	19.4% ^d^
	Another degree	3.6% ^a^	2.8% ^d^	4.2% ^d^	4.7% ^a^	4.9% ^d^	4.6% ^d^
**Occupation**	I don’t go to school yet	13.4%	15.8%	11.2%	9.2%	10.2%	8.3%
	Still go to school	28.0%	32.6%	23.9%	26.0%	28.6%	23.6%
	I am a trainee	4.8%	4.0%	5.5%	5.4%	4.4%	6.3%
	I am a student	8.2%	6.9%	9.4%	8.6%	8.0%	9.1%
	I am employed part-time	8.4%	2.9%	13.4%	8.4%	3.5%	12.8%
	I am employed full-time	24.9%	28.3%	22.0%	27.1%	31.2%	23.4%
	I am looking for work	1.6%	1.3%	2.0%	2.2%	2.1%	2.3%
	I am self-employed	1.5%	1.4%	1.6%	1.9%	1.6%	2.1%
	I am a pensioner	4.0%	3.5%	4.5%	5.8%	6.1%	5.4%
	Other	5.0%	3.4%	6.5%	5.5%	4.1%	6.7%

^A^ Lower level of education in secondary school in Germany also known as “Hauptschulabschluss”; ^B^ Higher level of education in secondary school in Germany also known as “Mittlere Reife/Realschulabschluss”; SD: standard deviation; * Reduced sample size (2 participants selected “diverse” when specifying their gender), as Survey 1 is an anonymous online survey, no reliable medical data on biological gender was available, so these two cases could not be included in the gender-specific analyses; ** A total of 6 people selected “diverse” when specifying their gender, in these cases, the biological gender stored in the NRAHF database was used for the statistical analyses to be carried out in order to avoid sample reduction; Survey 1 vs. Survey 2 total: ^a^ = *p* < 0.001; male vs. female within survey 1/2: ^d^ = *p* < 0.001.

**Table 2:** Medical and disease-related variables.

		Survey 1 (April 2020)			Survey 2 (April 2021)		
		Total N = 3558	Male n = 1670 *	Female n = 1886 *	Total N = 3179	Male n = 1505 **	Female n = 1674 **
**Severity of the congenital heart defect**							
Simple CHD		25.8% ^a^	20.4% ^d^	30.6% ^d^	14.2% ^a^	10.6% ^d^	17.5% ^d^
Moderate CHD		36.3% ^a^	37.7% ^d^	35.1% ^d^	39.1% ^a^	37.6% ^d^	40.5% ^d^
Complex CHD		30.5% ^a^	34.3% ^d^	27.1% ^d^	37.7% ^a^	43.5% ^d^	32.5% ^d^
Unclassified/unknown CHD		7.4% ^a^	7.6% ^d^	7.2% ^d^	8.9% ^a^	8.3% ^d^	9.5% ^d^
**Have you been to the doctor since the start of the COVID-19 pandemic? ^MA^**							
Cardiologists/pediatric cardiologists (practice)		24.4% ^a^	27.2% ^d^	21.8% ^d^	39.8% ^a^	42.6% ^e^	37.2% ^e^
Cardiologists/pediatric cardiologists (clinic/heart center)		23.1% ^a^	24.5%	21.9%	46.6% ^a^	47%	46.2%
Yes, with another doctor		54.8% ^a^	52.6% ^f^	56.7% ^f^	65.8% ^a^	63.6% ^f^	67.8% ^f^
No		22.7% ^a^	22.8%	22.6%	6.9% ^a^	6.7%	7%
**Already hospitalized for AHF since the beginning of the pandemic (cardiac catheterization/surgery)**		5% ^a^	5.3%	4.7%	14.3% ^a^	16.1% ^e^	12.7% ^e^
**Doctor’s appointment/check-up with pediatric cardiologist, cardiologist, heart clinic or hospital itself cancelled due to COVID-19**		8.0% ^c^	7.9%	8.1%	9.6% ^c^	8.7%	10.3%
**Cardiac catheter treatment cancelled even due to COVID-19**		0.4% ^c^	0.5%	0.2%	0.2% ^c^	1.1%	0.6%
**Operation itself cancelled due to COVID-19**		0.6%	1.0% ^f^	0.3% ^f^	0.9%	1.0%	0.9%
**Doctor’s appointment/check-up with pediatric cardiologist, cardiologist, heart clinic or hospital cancelled by doctor due to COVID-19**		9.7%	9.9%	9.5%	9.9%	9.3%	10.5%
**Cardiac catheter treatment cancelled by doctor due to COVID-19**		0.7%	0.7%	0.7%	1.0%	1.3%	0.8%
**Surgery cancelled by doctor due to COVID-19**		1.2%	1.3%	1.1%	1.6%	1.7%	1.6%
**If you have seen a doctor since the start of the COVID-19 pandemic, did the doctor talk to you/your parents about COVID-19 on their own initiative or did you or your parents ask about it?**							
Not been to the doctor since the start of the pandemic		22.7% ^a^	22.8%	22.6%	6.9% ^a^	6.7% ^f^	7.0% ^f^
Approached (without asking)		7.8% ^a^	7.5%	8.1%	17.8% ^a^	18.3% ^f^	17.3% ^f^
Received information material (without asking)		0.8% ^a^	0.7%	0.8%	1.7% ^a^	2.1% ^f^	1.3% ^f^
Approached + information material (without asking)		1.5% ^a^	1.7%	1.4%	3.4% ^a^	3.7% ^f^	3.2% ^f^
Conversation (upon request)		15.5% ^a^	16.8%	14.4%	27.6% ^a^	28.8% ^f^	26.5% ^f^
Information material (upon request)		0.6% ^a^	0.5%	0.6%	1.2% ^a^	1.5% ^f^	1.0% ^f^
Conversation + information material (upon request)		0.8% ^a^	0.9%	0.6%	2.5% ^a^	1.9% ^f^	3.0% ^f^
No		50.4% ^a^	49.1%	51.4%	39.0% ^a^	37.0% ^f^	40.7% ^f^
**Did you or your parents call your doctor because you or your parents wanted to know something about COVID-19 and your heart defect?**		21.6% ^a^	21.8%	21.3%	30.0% ^a^	29. 7%	30.3%
**Was your doctor able to answer the questions about COVID-19 and your heart defect?**							
Yes		25.2% ^a^	25.6%	24.8%	46.3% ^a^	49.6% ^e^	43.4% ^e^
No		8.9% ^a^	9.1%	8.7%	8.5% ^a^	7.7% ^e^	9.1% ^e^
Not yet spoken to the doctor about COVID-19		65.9% ^a^	65.3%	66.5%	45.2% ^a^	42.7% ^e^	47.5% ^e^
**Have you ever called a hotline/health authority about COVID-19?**							
Yes, I called and got through to someone		6.4% ^a^	6.8%	6.0%	21.1% ^a^	21.1%	21.1%
Yes. I called but never reached anyone		2.0% ^a^	1.6%	2.3%	3.7% ^a^	3.7%	3.8%
No. I did not call		91.6% ^a^	91.6%	91.6%	75.1% ^a^	75.3%	75.0%
**Have you been vaccinated against the flu?**							
Yes		50.9% ^a^	52.9% ^e^	49.2% ^e^	58.5% ^a^	61.1% ^e^	56.2% ^e^
No		46.7% ^a^	44.0% ^e^	49.0% ^e^	39.4% ^a^	36.4% ^e^	42.2% ^e^
I do not know		2.4% ^a^	3.1% ^e^	1.9% ^e^	2.0% ^a^	2.5% ^e^	1.7% ^e^
**Have you been vaccinated against pneumococcus?**							
Yes		53.7% ^c^	54.9%	52.6%	54.3% ^c^	55.4%	53.3%
No		28.7% ^c^	27.1%	30.0%	25.9% ^c^	24.9%	26.8%
I do not know		17.7% ^c^	18.0%	17.4%	19.8% ^c^	19.7%	19.8%
**Was COVID-19 the reason for the flu/pneumococcal vaccination/s?**							
Yes, COVID-19 was the reason		4.9% ^a^	4.7% ^f^	5.0% ^f^	9.7% ^a^	7.7% ^d^	11.5% ^d^
No, COVID-19 was not the reason		62.5% ^a^	64.2% ^f^	61.0% ^f^	61.4% ^a^	64.8% ^d^	58.4% ^d^
No, I was not vaccinated		28.6% ^a^	26.5% ^f^	30.4% ^f^	23.2% ^a^	21.8% ^d^	24.6% ^d^
I do not know		4.0% ^a^	4.6% ^f^	3.6% ^f^	5.6% ^a^	5.7% ^d^	5.6% ^d^
**Were there COVID-19 cases with family/acquaintances**		12.3% ^a^	11.8%	12.7%	63.5% ^a^	60.9% ^e^	65.9% ^e^
**I was infected with COVID-19 at least once**		0.4% ^a^	0.5%	0.3%	4.2% ^a^	3.9%	4.4%

CHD = congenital heart defects; ^MA^ Multiple answers were possible (unless the answer was “No”), so that results of more than 100% can be obtained when the individual values are added together; * reduced sample size (2 participants selected “diverse” when indicating their gender), as Survey 1 was an anonymous online survey, no reliable medical data on biological sex was available, so these two cases could not be included in the gender-specific analyses; ** A total of 6 people selected “diverse” when specifying their gender; in these cases, the biological gender stored in the NRCHD database was used for the statistical analyses to be carried out in order to avoid sample reduction; Survey 1 vs. Survey 2 total: ^a^ = *p* < 0.001/^c^ = *p* < 0.05; male vs. female within Survey 1/2: ^d^ = *p* < 0.001/^e^ = *p* < 0.01/^f^ = *p* < 0.05.

**Table 3:** Coronavirus knowledge.

		Survey 1 (April 2020)			Survey 2 (April 2021)		
		Total N = 3558	Patients n = 2029	Relatives n = 1529	Total N = 3179	Patients n = 2106	Relatives n = 1073
**I know what coronavirus is.**		6.4 ± 1.0 ^a^	6.5 ± 0.8 ^d^	6.3 ± 1.2 ^d^	6.3 ± 0.9 ^a^	6.3 ± 0.9	6.3 ± 1.0
**I am well informed about coronavirus.**		6.2 ± 1.0 ^a^	6.2 ± 0.9	6.1 ± 1.1	5.9 ± 1.0 ^a^	5.9 ± 1.1 ^d^	6.0 ± 1.0 ^d^
**I understand the media coverage (internet/TV/radio/newspaper) about coronavirus.**		6.1 ± 1.2 ^a^	6.4 ± 0.9 ^d^	6.0 ± 1.4 ^d^	5.8 ± 1.3 ^a^	5.8 ± 1.3	5.8 ± 1.3
**I trust the official information from the federal government, the federal authorities and the state authorities about coronavirus.**		5.5 ± 1.4 ^a^	5.6 ± 1.4 ^d^	5.4 ± 1.5 ^d^	4.9 ± 1.7 ^a^	5.0 ± 1.7 ^f^	4.8 ± 1.7 ^f^
**I also find out about coronavirus by sharing information on social networks (Facebook, YouTube, Instagram, WhatsApp, etc.).**		3.9 ± 2.1 ^a^	3.4 ± 2.1 ^f^	3.8 ± 2.2 ^f^	3.3 ± 2.1 ^a^	3.4 ± 2.1 ^e^	3.1 ± 2.1 ^e^
**I can tell which statements come from trustworthy sources and which are misinformation or “fake news”.**		5.6 ± 1.5 ^a^	5.8 ± 1.2 ^d^	5.3 ± 1.7 ^d^	5.4 ± 1.5 ^a^	5.6 ± 1.5 ^d^	5.2 ± 1.7 ^d^
**I talk to other people about coronavirus every day.**		5.4 ± 1.7 ^a^	5.4 ± 1.7 ^e^	5.3 ± 1.8 ^e^	5.1 ± 1.7 ^a^	5.0 ± 1.8	5.1 ± 1.9
**I follow the media coverage (internet/TV/radio/newspapers) about coronavirus every day.**		5.6 ± 1.7 ^a^	5.7 ± 1.6 ^e^	5.5 ± 1.8 ^e^	5.0 ± 1.9 ^a^	4.9 ± 1.9 ^e^	5.1 ± 1.9 ^e^
**I have changed my habits and everyday behavior because of coronavirus.**		5.9 ± 1.4 ^a^	5.8 ± 1.4 ^d^	6.0 ± 1.3 ^d^	5.7 ± 1.5 ^a^	5.7 ± 1.5	5.7 ± 1.5
**I feel restricted in my everyday life by the changes due to coronavirus.**		5.5 ± 1.5 ^a^	5.4 ± 1.5 ^d^	5.7 ± 1.4 ^d^	5.3 ± 1.7 ^a^	5.2 ± 1.8	5.3 ± 1.6
**Coronavirus hasn’t changed my life.**		3.0 ± 2.0 ^b^	3.01 ± 2.0	3.0 ± 2.1	2.9 ± 2.0 ^b^	2.8 ± 2.0	2.9 ± 2.1
**I’m worried about coronavirus.**		4.9 ± 1.6 ^c^	4.8 ± 1.6 ^d^	5.1 ± 1.6 ^d^	5.0 ± 1.6 ^c^	4.9 ± 1.6 ^d^	5.2 ± 1.6 ^d^
**I generally feel well informed about the heart defect by my doctor/my child’s doctor.**		5.4 ± 1.8 ^c^	5.3 ± 1.8 ^e^	5.5 ± 1.8 ^e^	5.3 ± 1.7 ^c^	5.1 ± 1.8 ^d^	5.7 ± 1.5 ^d^
**I understand what my doctor/my child’s doctor explains to me.**		6.0 ± 1.4 ^a^	5.9 ± 1.4 ^d^	6.2 ± 1.3 ^d^	5.9 ± 1.4 ^a^	5.7 ± 1.5 ^d^	6.2 ± 1.1 ^d^
**I trust my doctor/my child’s doctor.**		6.2 ± 1.1 ^a^	6.2 ± 1.2 ^d^	6.3 ± 1.0 ^d^	6.0 ± 1.3 ^a^	5.9 ± 1.4 ^d^	6.3 ± 1.0 ^d^
**I feel uncomfortable when I have to go to the doctor/when my child has to go to the doctor.**		3.4 ± 2.1 ^a^	3.2 ± 2.1 ^d^	3.6 ± 2.1 ^d^	3.0 ± 2.0 ^a^	3.0 ± 2.0 ^f^	3.1 ± 2.0 ^f^
**I try (with my child) to go to the doctor as rarely as possible.**		4.5 ± 2.1 ^a^	4.4 ± 2.1 ^f^	4.6 ± 2.1 ^f^	3.7 ± 2.2 ^a^	3.7 ± 2.2	3.8 ± 2.2
**I think it is important to be informed about coronavirus by my doctor/my child’s doctor.**		5.1 ± 1.9 ^b^	5.0 ± 1.9 ^d^	5.3 ± 1.8 ^d^	5.2 ± 1.7 ^b^	5.1 ± 1.8 ^d^	5.5 ± 1.7 ^d^
**If I don’t understand something about the heart defect and/or coronavirus or want to know something, I ask my doctor/my child’s doctor about it.**		5.2 ± 2.0 ^a^	4.8 ± 2.0 ^d^	5.7 ± 1.7 ^d^	5.5 ± 1.8 ^a^	5.3 ± 1.8 ^d^	6.0 ± 1.5 ^d^
**I feel well informed about coronavirus by my doctor/my child’s doctor.**		4.3 ± 2.1 ^a^	4.2 ± 2.0 ^d^	4.5 ± 2.1 ^d^	4.9 ± 1.8 ^a^	4.8 ± 1.8 ^d^	5.3 ± 1.7 ^d^
**I’m worried that I’m getting infected with coronavirus.**		4.6 ± 1.8 ^c^	4.4 ± 1.8 ^d^	4.7 ± 1.8 ^d^	4.5 ± 1.8 ^c^	4.4 ± 1.8	4.5 ± 1.9
**I will be infected with coronavirus in the next four weeks.**		2.8 ± 1.4 ^a^	2.8 ± 1.4 ^f^	2.7 ± 1.4 ^f^	2.3 ± 1.3 ^a^	2.3 ± 1.3	2.3 ± 1.3
**I know how I can protect myself from coronavirus infection.**		6.1 ± 1.3	6.0 ± 1.3 ^e^	6.1 ± 1.2 ^e^	6.1 ± 1.2	6.1 ± 1.3 ^f^	6.2 ± 1.2 ^f^
**I understand how coronavirus is spreading.**		6.4 ± 0.9 ^a^	6.4 ± 0.9	6.4 ± 1.0	6.3 ± 0.9 ^a^	6.3 ± 1.0 ^e^	6.4 ± 0.8 ^e^
**I know the symptoms of coronavirus.**		6.3 ± 1.0 ^a^	6.2 ± 0.9 ^f^	6.3 ± 1.0 ^f^	6.2 ± 1.0 ^a^	6.1 ± 1.0 ^f^	6.2 ± 0.9 ^f^

Assessment on a seven-point scale from 1 (not at all) to 7 (completely); mean ± standard deviation; Survey 1 vs. Survey 2 total: ^a^ = *p* < 0.001/^b^ = *p* < 0.01/^c^ = *p* < 0.05; Patients vs. Relatives within Survey 1/2: ^d^ = *p* < 0.001/^e^ = *p* < 0.01/^f^ = *p* < 0.05.

## Data Availability

Data cannot be shared for data protection reasons.

## Supplementary Materials

The following supporting information can be downloaded at: https://www.mdpi.com/article/10.3390/jcm14103462/s1, Table S1: Risk assessment.
