# Supplementary material for: Impact of the COVID-19 Pandemic on the Everyday Life and Healthcare of Patients with Congenital Heart Defects: Insights from Pandemic Onset to One Year Later
Source: J Clin Med. 2025 May 15;14(10):3462. doi: 10.3390/jcm14103462 (PMC12112097; doi:10.3390/jcm14103462)
Supplement: Supplementary file 1 [file jcm-14-03462-s001.zip › jcm-3566730-supplementary.pdf]

Journal of
Clinical Medicine

MDPI

Supplementary material

Table S1. Risk perception

		Survey 1 (April 2020)			Survey 2 (April 2021)		
		total N = 3,558	Patients n = 2,029	Relatives n = 1,529	total N = 3,179	Patients n = 2,106	Relatives n = 1,073
**The risk to my health is ...**	not present	1.6 %	2 %	1.1 %	0.9 %	1.1 %	0.7 %
	low	12.8 %	13.1 %	12.3 %	8.5 %	7.5 %	10.4 %
	rather low	29.8 %	27.2 %	33.2 %	23.2 %	18.9 %	31.7 %
	rather high	27.9 %	29.2 %	26.1%	28.6 %	29.5 %	26.8 %
	high	16.9 %	17.8 %	15.6 %	23.2 %	25.7 %	18.3 %
	very high	11 %	10.6 %	11.6 %	15.5 %	17.2 %	12.1 %
**The risk to my family's health is ...**	not present	1.7 %	1.8 %	1.6%	1.0 %	1.2 %	0.7 %
	low	12.0 %	11.0 %	13.2%	6.7 %	6.5 %	7.1 %
	rather low	35.4 %	33.9 %	37.5%	19.8 %	20.5 %	18.5 %
	rather high	30.1 %	31.9 %	27.7%	31.7 %	31.7 %	31.6 %
	high	14.7 %	15.2 %	14.0%	26.3 %	27.1 %	24.8 %
	very high	6.0 %	6.1 %	6.0%	14.5 %	13.0 %	17.3 %
**The risk to the health of my close personal environment (partner, close friends) is ...**	not present	5.7 %	5.0 %	6.7%	2.4 %	2.8 %	1.7 %
	low	19.7 %	22.5 %	16.0%	12.7 %	14.8 %	8.7 %
	rather low	39.2 %	38.0 %	40.7%	29.9 %	30.3 %	28.9 %
	rather high	22.2 %	21.3 %	23.3%	28.2 %	27.4 %	29.6 %
	high	9.5 %	9.3 %	9.7%	18.0 %	16.7 %	20.5 %
	very high	3.8 %	3.8 %	3.7%	8.9 %	8.0 %	10.6 %
**The risk to my social life is ...**	not present	2.6 %	3.0 %	2.2%	1.5 %	1.8 %	0.9 %
	low	13.7 %	15.1 %	11.9%	9.4 %	9.0 %	10.1 %
	rather low	29.3 %	30.2 %	28.3%	23.8 %	23.3 %	24.7 %
	rather high	27.2 %	26.6 %	28.1%	28.9 %	27.9 %	30.8 %
	high	18.1 %	17.2 %	19.3%	24.3 %	25.7 %	21.4 %
	very high	9.0 %	8.1 %	10.3%	12.2 %	12.3 %	12.0 %
**The risk to my family's social life is ...**	not present	3.1 %	4.0 %	1.9%	1.4 %	1.5 %	1.0 %
	low	15.1 %	16.1 %	13.9%	8.8 %	9.4 %	7.6 %
	rather low	33.1 %	34.0 %	32.0%	24.9 %	26.4 %	21.8 %
	rather high	28.6 %	28.0 %	29.3%	31.0 %	30.8 %	31.4 %
	high	14.2 %	13.4 %	15.4%	22.8 %	22.2 %	24.0 %
	very high	5.8 %	4.5 %	7.5%	11.2 %	9.7 %	14.1 %
**The risk to the social life of my close personal environment (partner, close friends) is ...**	not present	6.2 %	5.0 %	7.7%	2.5 %	2.9 %	1.7 %
	low	18.0 %	20.4 %	14.8%	10.6 %	11.4 %	9.1 %
	rather low	35.0 %	34.0 %	36.3%	27.4 %	27.4 %	27.5 %
	rather high	24.8 %	24.9 %	24.5%	29.8 %	29.5 %	30.2 %
	high	11.8 %	11.6 %	12.0%	20.7 %	20.5 %	21.2 %
	very high	4.4 %	4.1 %	4.7%	9.0 %	8.4 %	10.3 %
**The risk for my job, my studies or my vocational/school education is ...**	not present	18.5 %	15.8 %	22.0%	18.1 %	18.3 %	17.7 %
	low	15.0 %	17.2 %	12.0%	20.9 %	21.8 %	19.1 %
	rather low	20.6 %	21.3 %	19.6%	20.0 %	19.2 %	21.4 %
	rather high	20.2 %	18.5 %	22.4%	17.2 %	17.0 %	17.7 %

	high	14.6 %	14.8 %	14.3%	14.0 %	14.0 %	14.0 %
	very high	11.2 %	12.3 %	9.7%	9.8 %	9.6 %	10.1 %
**he risk for my family's job, studies or vocational/school education is ...**	not present	12.3 %	14.5 %	9.3%	11.2 %	12.9 %	7.9 %
	low	16.7 %	18.4 %	14.5%	16.6 %	18.7 %	12.5 %
	rather low	29.2 %	29.0 %	29.5%	25.4 %	26.4 %	23.2 %
	rather high	22.7 %	21.2 %	24.7%	22.0 %	20.8 %	24.2 %
	high	13.2 %	11.9 %	15.0%	16.2 %	14.2 %	20.2 %
	very high	5.9 %	5.0 %	7.1%	8.6 %	6.9 %	11.9 %
**The risk to the career, studies or vocational/school education of my close personal environment (partner, close friends) is ...**	not present	11.2 %	10.9 %	11.4%	9.6 %	10.6 %	7.5 %
	low	17.9 %	19.4 %	16.0%	14.2 %	15.9 %	10.8 %
	rather low	31.8 %	31.7 %	31.9%	28.3 %	28.1 %	28.8 %
	rather high	24.2 %	22.7 %	26.2%	24.5 %	23.6 %	26.3 %
	high	10.6 %	10.8 %	10.3%	16.3 %	15.3 %	18.3 %
	very high	4.3 %	4.4 %	4.2%	7.1 %	6.6 %	8.3 %
**The risk for the economy/working life is ...**	not present	1.9 %	1.4 %	2.6 %	1.3 %	1.4 %	1.2 %
	low	2.0 %	2.2 %	1.8 %	2.5 %	2.8 %	1.9 %
	rather low	4.2 %	4.2 %	4.1 %	7.0 %	7.2 %	6.7 %
	rather high	20.6 %	19.4 %	22.1 %	24.1 %	24.2 %	24.0 %
	high	32.9 %	43.4 %	31.0 %	34.5 %	35.2 %	33.0 %
	very high	38.4 %	38.3 %	38.5 %	30.6 %	29.2 %	33.3 %
**The risk to the healthcare system is ...**	not present	1.7 %	1.3 %	2.2 %	1.7 %	1.9 %	1.4 %
	low	3.6 %	3.6 %	3.5 %	3.2 %	3.4 %	2.7 %
	rather low	10.6 %	10.0 %	11.4 %	8.5 %	8.2 %	9.1 %
	rather high	34.9 %	33.9 %	36.2 %	27.3 %	26.7 %	28.6 %
	high	31.2 %	32.6 %	29.3 %	33.2 %	33.0 %	33.5 %
	very high	18.1 %	18.5 %	17.5 %	26.1 %	26.9 %	24.7 %
**The risk to social cohesion is ...**	not present	2.0 %	2.1 %	1.8 %	0.9 %	1.0 %	0.7 %
	low	11.2 %	11.6 %	10.7 %	3.5 %	3.8 %	3.0 %
	rather low	25.9 %	26.2 %	25.6 %	11.1 %	11.4 %	10.3 %
	rather high	31.4 %	30.8 %	32.2 %	26.8 %	25.6 %	29.2 %
	high	20.7 %	21.6 %	19.4 %	32.9 %	33.8 %	31.2 %
	very high	8.8 %	7.7 %	10.2 %	24.8 %	24.4 %	25.5 %
**The risk to cultural life is ...**	not present	2.0 %	1.8 %	2.2 %	1.0 %	0.8 %	1.4 %
	low	4.6 %	4.9 %	4.1 %	2.5 %	2.8 %	2.0 %
	rather low	8.9 %	9.1 %	8.6 %	5.4 %	5.7 %	4.7 %
	rather high	24.7 %	25.1 %	24.2 %	16.3 %	16.2 %	16.4 %
	high	30.8 %	30.4 %	31.3 %	29.5 %	30.1 %	28.3 %
	very high	29.1 %	28.7 %	29.5 %	45.4 %	44.4 %	47.3 %

Survey 1 vs. Survey 2 total: $p < .001$ / $p < .01$ / $p < .05$; Patients vs. Relatives within Survey 1/2: $p < .001$ / $p < .01$ / $p < .05$
